# Sclerotherapy of Intraoral Superficial Hemangioma

**DOI:** 10.1155/2016/4320102

**Published:** 2016-11-23

**Authors:** Resmije Ademi Abdyli, Yll Abdyli, Feriall Perjuci, Ali Gashi, Zana Agani, Jehona Ahmedi

**Affiliations:** ^1^Department of Oral Surgery, Medical Faculty, University of Prishtina, Dental Branch, 10000 Prishtina, Kosovo; ^2^Medical Faculty, University of Prishtina, Dental Branch, 10000 Prishtina, Kosovo

## Abstract

Hemangioma is the clinical term for a benign vascular neoplasm due to proliferation of the endothelial lining of blood vessels. Their most frequent location is the body skin and oral mucosa. One of the treatment modalities for hemangiomas is intralesional injection of sclerosing agents which cause the damage of blood vessels followed by their obliteration. The objective of the study was to describe the facility of application and evaluate the efficiency of sclerotherapy with aethoxysklerol 1%.* Method*. The case presented with intraoral submucosal hemangioma of the cheek was treated by intralesional injection of aethoxysklerol 3% diluted in water for injections at a 4 : 1 ratio (0.75%) at the first appointment and 3 : 1 (1%) at the second appointment. The effect of sclerotherapy was evaluated on the following visits in time intervals of two weeks.* Results*. The hemangioma disappeared without complications after the second injection of aethoxysklerol 1%. The successful results of the study were comparable to the data of literature with variations according to the used sclerosant agent, its concentration, the number of injections, and the intervals between each session.* Conclusion*. Since sclerotherapy is a very effective, inexpensive, and easy-to-apply treatment, it should be the treatment of choice, especially for intraoral superficial hemangiomas.

## 1. Introduction

Hemangioma is a vascular neoplasm or a vascular anomaly due to proliferation of blood vessels. They occur anywhere in the body, but skin and oral mucosa in the region of the lips, tongue, and buccal mucosa are most commonly affected. Therefore, the dentist or oral surgeon should be informed about their clinical aspect, diagnosis, and therapy [1–3].

The clinical aspect of oral hemangiomas depends on their location and depth. Usually hemangiomas present as blood-filled asymptomatic swellings or red/bluish-purple discolorations, but their progressive growth can increase the likelihood of local traumatic injuries followed by unexpected bleeding [4–7]. The size of hemangiomas is variable, ranging from a few millimeters to several centimeters in the form of a macule, papule, nodule, or tumor, with elastic or fibrous consistency [8].

In order to obtain a definite diagnosis of vascular malformations (of suspected hemangioma), different clinical examination methods can be implemented, including digital compression and diascopy [6, 7] and other supplementary imaging tests such as ultrasonography with Doppler and MRI [9, 10].

Various modalities have been used in the treatment of hemangiomas, depending on their location, size and depth, evolution of injury, and involvement of adjacent structures [8, 10].

The gold standard for hemangioma treatment, especially for smaller circumscribed lesions and peripheral hemangiomas, is conventional surgical excision [3, 11]. However, complications that arise from conventional invasive surgical procedures such as excessive postoperative bleeding compelled the use of other different therapeutic alternatives including systemic corticosteroids, laser therapy, cauterization, cryotherapy, radiotherapy, and sclerotherapy [4, 12–14]. These modes of treatment can be applied individually or in concert.

*The Objective*. The Objective of the study was to describe the facility of the application of sclerosing agents and evaluate the effectiveness of sclerotherapy with aethoxysklerol 1% as one of the treatment options of oral superficial hemangioma.

## 2. Case Description and Treatment Method

**Figure 1:**
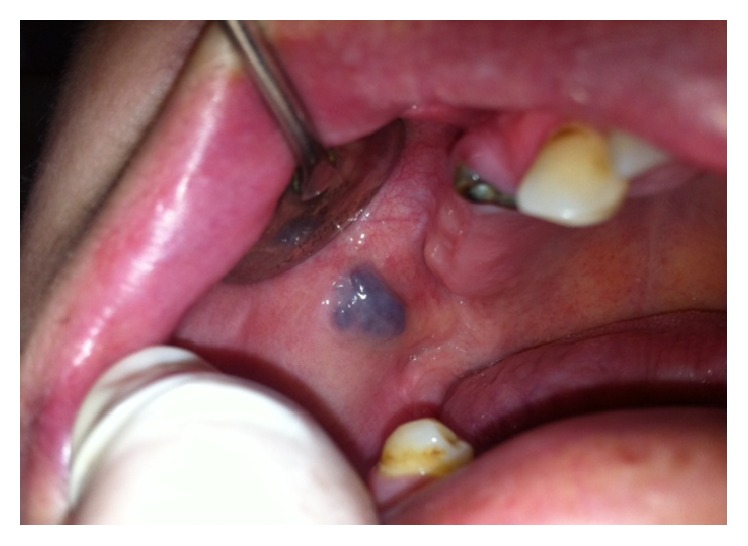
Submucosal hemangioma of the cheek at the right molar region.

A 53-year-old female was referred to the Department of Oral Surgery at UDCCK, Dental Branch of Medical Faculty, University of Prishtina, Kosovo, for surgical treatment of an intraoral submucosal lesion. According to patient's complaint, she had noticed an asymptomatic blue-colored lesion on the right side of the cheek about six months ago. Intraoral physical examination revealed an indolent, well defined purple colored lesion under intact mucosa of the cheek, soft on palpation with dimensions around 1.5 × 0.8 cm (Figure 1). Based on medical history and clinical examination, the lesion was diagnosed as submucosal hemangioma of the cheek.

Considering the superficial location of the lesion, the decision was, in lieu of surgical treatment, to opt for sclerotherapy with aethoxysklerol 1% applied on 3-4 (three to four sessions) at intervals of 10–14 days, depending on the lesion's treatment progress. Taking into account the fact that we had only 3% aethoxysklerol at our disposal, this sclerosing agent was diluted with normal saline to desired concentration prior to intralesional injection.

**Figure 2:**
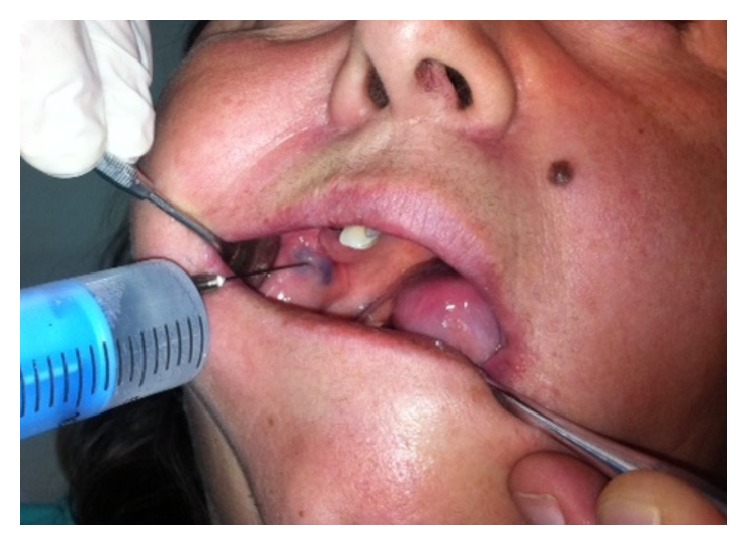
First intralesional injection of aethoxysklerol 0.75%.

Owing to the doubt that aethoxysklerol 1% can cause tissue damage at the injection site after intralesional injection, the available aethoxysklerol 3% was diluted with normal saline at a 1 : 4 ratio, obtaining 0.75% concentration of sclerosing agent. Slow injection of 1–1.5 mL aethoxysklerol 0.75% was performed without anesthesia into the lumen of the lesion (Figure 2). After injecting the agent, local hemostasis was performed by digital compression at the site of injection. The effect of therapy was evaluated on the following visits.

**Figure 3:**
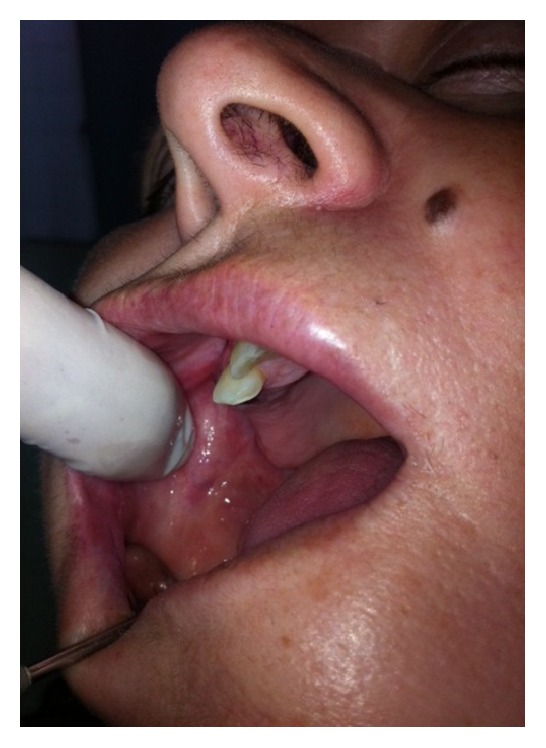
Second appointment after twelve days: regression of hemangioma.

**Figure 4:**
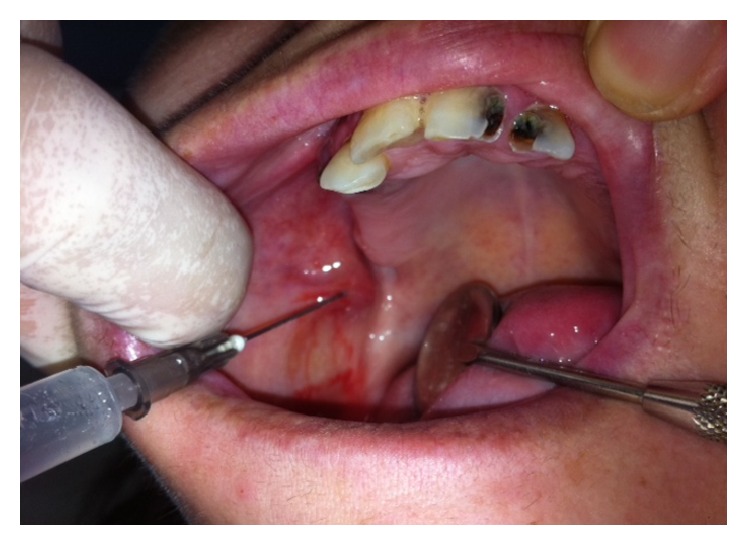
Second intralesional injection of aethoxysklerol 1%.

At the next visit, appointed after twelve days (Figure 3), the size of the lesion was reduced dramatically, necessitating only one other subsequent intralesional injection of 1 mL aethoxysklerol solution of 1% concentration, obtained by dilution of aethoxysklerol 3% at normal saline at a ratio of 3 : 1 (Figure 4).

**Figure 5:**
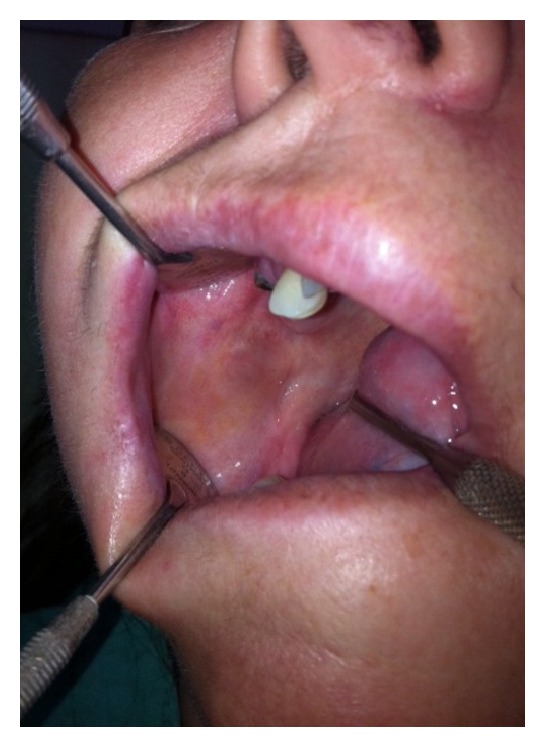
Third following visit: hemangioma disappeared without complications.

During the following third visit (Figure 5), around two weeks later, intraoral examination revealed complete disappearance of the lesion, so the sclerosant therapy was terminated and the patient was appointed for the next visit after one month. After almost two months, by a phone call, the patient notified us that the lesion had completely disappeared without any sign of recurrence.

## 3. Discussion

There are many treatment modalities reported in the literature for oral hemangiomas, such as intralesional and systemic corticosteroid treatment, surgical excision, thermocauterization, laser photocoagulation, and sclerotherapy [4, 12–14]. Each of the treatment modalities has its own risks and advantages.

Advantages of sclerotherapy to other hemangioma treatment modalities include it being very simple and safe to apply, affordable, and readily available, with most of this being due to not requiring special equipment for application and having no need for hospitalization of the patient.

Most importantly, it has shown high efficacy, offering partial or complete regression of the lesion without bleeding [4, 15–18]. Disadvantages of sclerotherapy include postoperative pain and burning sensation, potential anaphylactic reaction, tissue necrosis, and airway compromise [19].

Currently, sclerotherapy is largely employed because of its effectiveness and ability to conserve the surrounding tissues [20]. Sclerotherapy has been proven effective in the treatment of benign vascular lesions, especially small lesions located on sites with esthetic impact, where surgery could leave unpleasant scarring [4, 15, 16, 21, 22].

Frequently used sclerosing agents are sodium morrhuate, sodium psylliate, hypertonic glucose solution, sodium tetradecyl sulfate, ethanolamine oleate, and polidocanol (aethoxysklerol 3%, 1% or 0.5%) [3, 4, 13, 15].

One of the sclerosing agents used for many years in the treatment of hemangioma and varicose veins is polidocanol (aethoxysklerol 3%, 1%, or 0.5%) [13, 15, 16, 18, 23–28].

Polidocanol (aethoxysklerol) and sodium tetradecyl sulfate (STS) are the best known detergent solutions which act by causing localized inflammatory reaction, obliterative thrombosis of hemangiomatous space, and subsequent fibrosis of the endothelial spaces, leading to the regression of the lesion [25–27]. These advantages of sclerosant use are the absence of pain on intravascular injection, a high level of efficacy and safety, and a very low occurrence rate of allergic reactions [28].

The quantity of injected sclerosing agents and the number of applications during the sclerotherapy treatment depend on the size and location of the lesion and involvement of adjacent structures, not forgetting to mention the obtained results, which should be evaluated before the administration of the next dose after an interval of 1 to 2 weeks [4, 14, 15, 26].

The treatment employed in the presented case was sclerotherapy with aethoxysklerol 1%. The concentration of the sclerosing agent (1%), number of treatment sessions (3-4), and intervals between each session (12 to 14 days) were planned based on our previous experience with hemangioma treatment by aethoxysklerol 1%. Due to the fact that we only had aethoxysklerol 3% at our disposal, the 3% solution was diluted in normal saline in a ratio of 1 : 4 obtaining aethoxysklerol 0.75% for intralesional injection on the first session. On the second session after twelve days, the agent was diluted 1: 3 (1%) obtaining aethoxysklerol 1%. The results of sclerotherapy were followed and assessed after a certain time period (ten days–two weeks) from the sclerotherapy session. The case has proven that intralesional injection of the aethoxysklerol 0.75%–1% was very effective, inducing rapid regression of the lesion after the second intralesional injection; therefore, it was considered that two sessions of injections were sufficient for the treatment of this superficial hemangioma.

The results of the actual study were similar to the data of literature relating to sclerotherapy, with variations according to the type of sclerosing agent, its concentration, the number of injections, and the intervals between each treatment session [13, 14, 18, 24, 29, 30].

Winter et al. in 2000 also published their experience with polidocanol in 132 patients with cavernous hemangiomas, demonstrating a satisfactory response and requiring only one to three injections [18].

Another treatment option of oral hemangioma treatment is laser therapy based on the coagulative effect of superpulsed laser beams, leading to a virtually painless vaporization of tissue [31]. Lasers have indications for use in dentistry for incision, excision, and coagulation of intraoral soft tissue. They are well suited for surgical removal of intraoral hemangiomas because they offer a bloodless operational technique and avoid tissue damage. Advantages of laser therapy include minimal postoperative pain, minimally invasive surgery, and no need for sutures with no intraoperative or postoperative adverse effects [32, 33].

Laser treatment is currently used for thin, superficial lesions, ulcerated hemangiomas, and residual erythema and telangiectasias. Several lasers are used for hemangioma treatment, such as the pulsed dye laser (PDL), Nd:YAG laser, the KTP and the CO_2_, and Erbium lasers [31–34].

The effective depth of penetration of PDL is minimal to a depth of around 1.2 mm; therefore, it is not very effective in treating deeper hemangiomas, which may continue to grow even if the superficial component recedes [34].

Nd:YAG laser is used for treating the deep component of hemangiomas of the oral cavity and requires very careful use by experienced physicians.

KTP lasers are also an option, especially for deeper, thicker lesions. The KTP laser is actually a type of Nd:YAG laser (1064 nm) that is modified when the 1064 nm light is passed through a KTP crystal. When the KTP laser is used with an intralesional bare fiber, the laser light is sent directly into the deep component of the hemangioma, delivering the maximum amount of laser energy to this section while limiting cutaneous damage [35]. This allows better lesion penetration than a PDL laser but it carries less risk of scarring than an Nd:YAG laser [36].

Currently, there are no optimal laser systems for hemangioma treatment [37].

Crisan et al. in 2010 confirmed that laser therapy in the treatment of vascular lesions was more effective than the sclerotherapy procedure [35], while Witman et al in 2006 revealed complications from PDL treatment of hemangiomas, including ulceration, pain, residual scarring components of hemangiomas, and in one instance life-threatening bleeding [38]. Therefore, laser and cryotherapy are not commonly used in treatment of haemangiomas due to scarring or hyperpigmentation, skin atrophy, and slight depression of the skin and due to high cost [39, 40].

The surgical treatment of oral hemangiomas, similar to other treatment modalities, has its own risks and advantages.

The advantage of the surgical treatment is that, unlike other forms of hemangioma treatment, it allows for a microscopic diagnosis. In addition, the complete surgical excision of these lesions offers the best chance of cure, but it is often accompanied with the risk of excessive postoperative bleeding and severe functional impairment of vital functions, such as swallowing, speech, and airway maintenance. Therefore, surgical intervention as a treatment modality for haemangioma is considered a last resort due to intraoperative bleeding, postoperative scarring, incomplete excision, recurrence, functional impairment, and surgical morbidity [15, 41].

## 4. Conclusion

Since sclerotherapy is a very effective, inexpensive, and easy-to-apply treatment modality, it should be regarded as the primary choice of treatment, especially for intraoral superficial hemangiomas.
